# Cutthroat Trout Virus—Towards a Virus Model to Support Hepatitis E Research

**DOI:** 10.3390/v8100289

**Published:** 2016-10-20

**Authors:** Marcus von Nordheim, Michel Boinay, Remo Leisi, Christoph Kempf, Carlos Ros

**Affiliations:** 1 Department of Chemistry and Biochemistry, University of Bern, Bern 3012, Switzerland; michel.boinay@students.unibe.ch (M.B.); remo.leisi@dcb.unibe.ch (R.L.); christoph.kempf@dcb.unibe.ch (C.K.); carlos.ros@dcb.unibe.ch (C.R.); 2 CSL Behring AG, Bern 3014, Switzerland

**Keywords:** cutthroat trout virus, hepatitis E virus, virus model

## Abstract

Cutthroat trout virus (CTV) is a non-pathogenic fish virus belonging to the *Hepeviridae* family, and it is distantly related to hepatitis E virus (HEV). Here, we report the development of an efficient cell culture system where CTV can consistently replicate to titers never observed before with a hepevirus. By using the rainbow trout gill (RTGill-W1) cell line, CTV reaches 10^10^ geq/mL intracellularly and 10^9^ geq/mL extracellularly within 5–6 days in culture. We additionally established a qPCR system to investigate CTV infectivity, and developed a specific antibody directed against the viral capsid protein encoded by ORF2. With these methods, we were able to follow the progressive accumulation of viral RNA and the capsid protein, and their intracellular distribution during virus replication. Virus progeny purified through iodixanol density gradients indicated—that similar to HEV—CTV produced in cell culture is also lipid-associated. The lack of an efficient cell culture system has greatly impeded studies with HEV, a major human pathogen that causes hepatitis worldwide. Although several cell culture systems have recently been established, the replication efficiency of HEV is not robust enough to allow studies on different aspects of the virus replication cycle. Therefore, a surrogate virus that can replicate easily and efficiently in cultured cells would be helpful to boost research studies with hepeviruses. Due to its similarities, but also its key differences to HEV, CTV represents a promising tool to elucidate aspects of the replication cycle of *Hepeviridae* in general, and HEV in particular.

## 1. Introduction

Cutthroat trout virus (CTV) was first isolated from spawning adult trout in 1988 [1]. Based on the similarities to hepatitis E virus (HEV), CTV was classified as a member of the *Hepeviridae* family [2]. Recently, this family has been divided into two proposed genera, *Orthohepevirus* (all mammalian and avian HEV isolates) and *Piscihepevirus* (CTV) [3]. The genome of CTV is a positive sense, single-stranded RNA molecule that is 7.2 kb in length, consisting of three open reading frames and ending in a poly A tail. Upon comparing the genome organization with other hepeviruses, it was deduced that ORF1 encodes a polyprotein for viral replication, and that ORF2 encodes the capsid protein [2]. It is likely that ORF3 encodes a phosphoprotein, which in HEV is required for budding and for the formation of lipid-associated progeny particles, which are observed in serum and cell culture supernatant (SN) [4]. The location of ORF3 is similar to that of HEV genotype 4, where its 5′ end does not overlap with ORF1. Upon pairwise alignment with HEV, it was shown that the nucleotide sequence identity of the 5′ UTR is 44%, and that that of the 3′ UTR is 40%. The amino acid identities of ORF1, ORF2, and ORF3 are 26%, 19%, and 13%, respectively [2]. The genome of CTV is therefore similar in size and organization to that of HEV. CTV has been propagated in CHSE-214 cells [1,2,5,6], with viral titers reaching between 10^7^ and 10^8^ geq/mL after 20 days of infection [6]. Being similar to HEV, non-pathogenic to humans, and able to replicate in cultured cells, CTV has been proposed as a promising model virus for HEV [2,6].

HEV was first encountered in 1978 [7], and represents the leading cause of acute hepatitis in the world [8]. It is responsible for epidemics in developing countries, and occurs in endemic form in industrialized countries [9]. Even though most cases of acute HEV are self-limited, chronic infections can occur in immunocompromised patients [10,11]. For unknown reasons, the case fatality rate among infected pregnant women is very high, reaching 10%–30% [12]. It is not clear why pregnant women are at greater risk, but changes in hormonal levels during pregnancy and their effect on the immune system are thought to be involved [11].

The efficient propagation of HEV in cell culture is critical for detailed study of different steps of the replication cycle, such as cell attachment, uptake, uncoating, and egress. Many attempts have been made to efficiently replicate HEV in vitro. Different cell lines have been tested, including human embryo lung diploid cells (2BS) [13], human hepatoma cells (PLC/PRF/5) [14,15], and human lung cancer cells (A549) [15,16,17]. Furthermore, animal models [18] and infectious clones [19] were developed with the aim of gaining insight into HEV pathogenesis and to improve HEV replication. Even though some cell culture systems have been established with variable success [14,15,20,21,22], their moderate efficiency in terms of titer levels and culture time remains a major drawback, complicating the studies on HEV. For this reason, many basic aspects of HEV replication remain unknown. Hence, a surrogate model for HEV that can efficiently replicate in cell culture is greatly needed.

In the present studies, we describe the development of a cell culture system where CTV replicates with an efficiency never before observed with HEV or with any other member of the *Hepeviridae* family, and the establishment of analytical tools to characterize the infection. The analysis of the virus progeny revealed that—similar to HEV—CTV exhibits the same intriguing characteristic of possessing an envelope after being released into the cell culture SN. Using the non-pathogenic CTV as a virus model for HEV would not only allow HEV research to be tackled from a different angle, but would also eliminate safety issues associated with HEV research.

## 2. Materials and Methods

### 2.1. Cell Culture and Virus Propagation

The rainbow trout gill (RTGill-W1) cell line and the rainbow trout liver (RTL-W1) cell line were a gift from Sylvie Bony from the University of Lyon, France. CTV (Heenan88 isolate) was kindly provided by Yannik Debing from the Rega Institute for Medical Research, Department of Microbiology and Immunology, Leuven, Belgium. The cells were maintained in Leibovitz’s L-15 media (Thermo Fisher Scientific, Waltham, MA, USA) supplemented with 10% heat-inactivated fetal calf serum (Amimed, London, UK) and 50 U/mL penicillin/streptomycin (Biochrom/Millipore, Berlin, Germany) at 18 °C. For virus infection, 4 × 10^4^ cells were added in 200 µL of medium to a 96-well plate (TPP, Trasadingen, Switzerland). Once confluent, the cells were inoculated overnight with 4 × 10^6^ genome equivalents in 50 µL Minimal Essential Media (MEM) (Sigma-Aldrich, St. Louis, MO, USA) supplemented with 10 mM HEPES (4-(2-hydroxyethyl)-1-piperazineethanesulfonic acid), 2% heat-inactivated fetal calf serum, and 50 U/mL penicillin/streptomycin. At 5 h post-infection, the cells were washed three times with MEM and then incubated in MEM at 18 °C over a period of several weeks.

### 2.2. Quantification of CTV RNA by Reverse Transcription-Quantitative Polymerase Chain Reaction

An RNA standard was constructed by initially reverse transcribing the CTV RNA. Subsequently, a 349 nucleotide-long sequence from the CTV ORF1 helicase region between nucleotides 3217 and 3565 was amplified and cloned into a pDrive vector (Qiagen, Düsseldorf, Germany). Following transformation into XL10 Gold cells, the plasmid containing the specific insert was isolated and linearized, and the region of interest was transcribed using a T7 High Yield RNA Synthesis Kit (New England Biolabs, Ipswich, MA, USA). Prior to quantification, CTV RNA was extracted using the QIA-amp Viral RNA Kit (Qiagen). The extracted samples were prepared for quantification using the iTaq Universal One-Step RT-qPCR Kit (Bio-Rad, Hercules, CA, USA). The primers used amplify the genomic region between nucleotides 3247 and 3409: (Forward) 5′-ggcaaccatcctctacaaacac-3′ and (Reverse) 5′-gatgtcttgtgggagcctgtag-3′. The thermal cycling conditions were 50 °C for 10 min, 95 °C for 1 min, and 40 cycles of 95 °C for 10 s, 65 °C for 20 s, and 72 °C for 40 s.

### 2.3. Infectivity Assay

For virus titration, an infectivity assay based on CTV RNA quantification was developed. Columns 2–11 of two 96 well plates were seeded with 4 × 10^4^ RTGill-W1 cells in Leibovitz’s L-15 media. One week later, serial dilutions of cell culture supernatant containing CTV (4 × 10^6^ genome copies/µL) were added in 8-plicates (50 µL in 2% MEM per well). The starting dilution was 10**^−^**^2^, with a serial dilution factor of five. One day after the infection, the media was removed, the cells were washed, and 200 µL of fresh 2% MEM was added per well. After two weeks, the plates were frozen to −70 °C to lyse the cells. RNA was extracted from samples (140 µL) by using the QIAamp viral RNA kit (QIAGEN) and quantified as specified above. The infectious titer was determined by the Spearman–Kärber method and expressed as log TCID_50_/mL.

### 2.4. Antibodies

The genetic sequence of the CTV ORF2 region—encoding residues 345–634 of the capsid protein—was cloned into pTAC-MAT-Tag-2 expression vector (Sigma-Aldrich). Protein expression was performed in BL-21 Gold (DE3) competent cells (Agilent Technologies, Santa Clara, CA, USA). Inclusion bodies were isolated and then solubilized in lysis buffer containing 100 mM NaH_2_PO_4_, 10 mM Tris-Cl, and 8 M urea at pH 8.0. The recombinant (rec.) capsid protein was purified with Ni-NTA Agarose beads (Qiagen, Hilden, Germany) and then dialyzed against refolding buffer to remove the urea. The purified protein (approximately 1 mg/mL) was used to raise a polyclonal antibody in a rabbit by immunoGlobe (Himmelstadt, Germany). The mature and purified primary polyclonal antibody was obtained at a concentration of 1.18 mg/mL in PBS.

The mouse monoclonal antibody J2 against dsRNA was purchased from English and Scientific Consulting (Szirák, Hungary). Secondary antibodies used for immunofluorescent microscopy included the goat anti-rabbit IgG (H + L) Alexa Fluor 488 and the goat anti-mouse IgG (H + L) Alexa Fluor 546, both purchased from Thermo Fisher Scientific.

### 2.5. Dot Blot

Serial dilutions of the purified recombinant capsid protein were spotted onto a Nitrocellulose Membrane, 0.45 µm (Bio-Rad). As a negative control, the VP1u-MAT protein from parvovirus B19 was used. The same procedure as for Western blotting was used for antibody incubation and visualization.

### 2.6. Western Blotting

A six well plate (TPP) with confluent RTGill-W1 cells was inoculated with 2 × 10^8^ genome equivalents in 3 mL MEM per well. At 5 h post-infection, the medium was removed, and the cells were washed three times with 1 mL MEM and further incubated in 3 mL MEM. At increasing days post-infection, the SN was removed for later analysis, and the cells were trypsinized and washed once with PBS to remove uninternalized viruses. All samples were resuspended in 100 µL PBS and subjected to two freeze–thaw cycles. Cell debris was removed by centrifugation, and 10 µL of the SN was mixed with 10 µL of 2× Laemmli sample buffer. Proteins in the samples were separated by SDS-PAGE on a NuPAGE 4%–12% Bis-Tris Gel (Invitrogen, Carlsbad, CA, USA) and then blotted onto a polyvinylidene difluoride membrane (0.45 µm, Millipore, Billerica, MA, USA). The membrane was incubated with anti-CTV ORF2 pAb, washed with TBST (Tris-buffered saline, 0.1% Tween 20), and then incubated with polyclonal goat anti-rabbit immunoglobulins conjugated with HRP (Dako/Agilent Technologies). After final washings with TBST, the proteins were visualized by using SuperSignal West Dura Extended Duration Substrate (Thermo Fisher Scientific).

### 2.7. Immunofluorescence

Cells grown in a 12-well plate (TPP) on collagen-coated cover slips (Neuvitro, Vancouver, WA, USA) were fixed and permeabilized with acetone/methanol (50:50) for 7 min at −20 °C. The fixative was removed, and the cells were allowed to dry. For blocking purposes, PBS containing 20% (*v*/*v*) goat serum (Dako/Agilent Technologies) was added to the cells for 40 min and then labeled with primary antibodies in a humidifying chamber for 1 h. After PBS washes for 1 h, the cells were labeled with Alexa Fluor conjugated secondary antibodies for 1 h. After PBS washes for 1 h, the cells were labeled with DAPI (4’,6-diamidino-2-phenylindole) to reveal their nuclei and allowed to dry after treating with ethanol. The samples were mounted with Mowiol (Calbiochem, La Jolla, CA, USA) containing 30 mg/mL of Dabco (Sigma) as an antifading agent. The cells were examined with a Zeiss LSM 880 with Airyscan confocal microscope equipped with Plan-Apochromat 20×/0.8 M27 and Plan-Apochromat 63×/1.4 Oil DIC M27 objective lenses.

### 2.8. Iodixanol Density Gradient Centrifugation

A density gradient was prepared in Ultra-Clear tubes (14 × 89 mm, Beckman Coulter, Brea, CA, USA) ranging from 0 to 48% (*w*/*w*) OptiPrep Density Gradient Medium (Sigma-Aldrich) in TBS buffer supplemented with 0.5% (*w*/*v*) BSA. Cell culture SN treated with or without 1% NP40 (Applichem, Darmstadt, Deutschland) was layered onto the gradient and then centrifuged using a SW-41 Ti rotor (Beckman Coulter) at 200,000× *g* at 5 °C for 6 h in an Optima XPN-80 ultracentrifuge (Beckman Coulter). Fractions of 340 µL were collected from the top. The density of each fraction was measured by refractometry, and the RNA concentration was quantitated by one-step RT-PCR after RNA extraction.

## 3. Results

### 3.1. CTV Replication in Cell Culture

**Figure 1:**
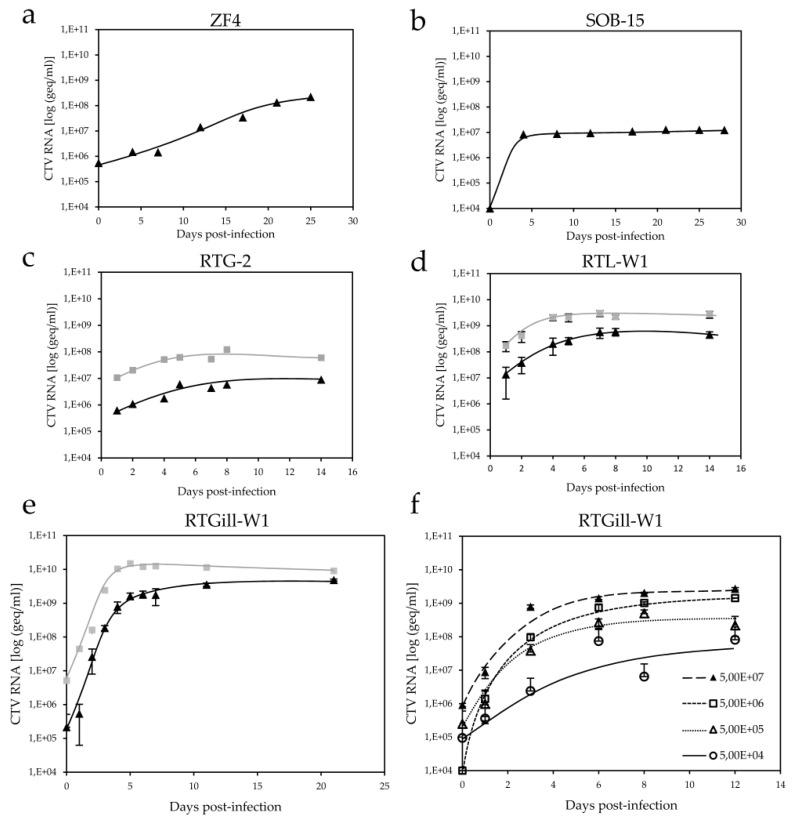
Quantitative analysis of cutthroat trout virus (CTV) RNA in cell culture supernatant (SN) (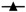) and cellular fractions (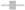) by reverse transcription-polymerase chain reaction (RT-PCR). (**a**) ZF4; (**b**) SOB-15; (**c**) RTG-2; (**d**) duplicate samples of RTL-W1; and (**e**) triplicate samples of RTGill-W1 cells; (**f**) Comparison of viral RNA in duplicates in cell culture SN using 10-fold dilutions of input CTV, as indicated.

Previous studies have shown that CTV can replicate in CHSE-214 cells; however, the efficiency was comparable to that described for HEV with certain cell lines [23]. With the aim of finding a better cell line where CTV could replicate to high titers with short incubation times, virus propagation was examined in ZF4, SOB-15, RTG-2, RTGill-W1, and RTL-W1 cell lines over a period of several weeks. From all cell lines tested, CTV replicated with the highest efficiency in RTGill-W1 (Figure 1e) and RTL-W1 cells (Figure 1d), and with the lowest efficiency in SOB-15 cells (Figure 1b). Unlike previous observations, where a diffuse type of cytopathic effect (CPE) was described in infected CHSE-215 cells [2], no clear CPE was observed in any of the tested cell lines. The virus progeny released into the cell culture SN of RTGill-W1 cells was already detectable at 1 day post-infection (dpi), and reached over 10^9^ geq/mL after only 5 dpi, followed by a plateau where no significant replication occurred. A similar trend could be observed from the cellular fraction, which reached 1.48 × 10^10^ geq/mL on day 5 in equal volume as cell SN. The cellular fraction should contain both progeny and naked viral RNA, which may account for the increased RNA levels. In contrast, RNA values from the cell culture SN exclusively reflect nuclease-resistant RNA, since extracted CTV RNA incubated in cell culture SN was readily digested by endogenous RNases and became undetectable by RT-PCR. Figure 1d shows very similar replication kinetics in RTL-W1 cells, reaching a plateau after 6 dpi, with a slightly lower maximum of 5 × 10^8^ geq/mL in the SN and 3 × 10^9^ geq/mL in the cellular fraction. In both cell lines, the amount of viral RNA in the cellular fraction exceeded the amount in the SN by about one log. As expected, CTV replication kinetics were influenced by the amount of input virus (Figure 1f).

**Figure 2:**
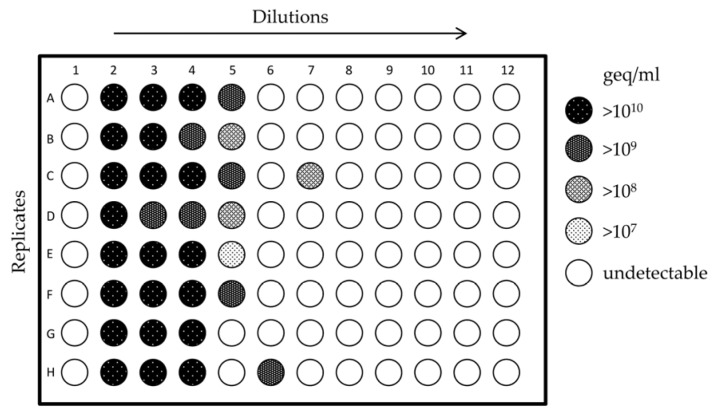
Determination of cutthroat trout virus (CTV) infectious titer by the Spearman–Kärber method. A 96-well plate used to determine the infectious titer of CTV is shown. Serial five-fold dilutions in 8-plicates—starting from a 10^−2^ dilution of CTV cell culture supernatant (SN)—were used to infect RTGill-W1 cells (seeded 4 × 10^4^ cells per well). Rows 1 and 12 were left blank. The infectious titer of the stock virus SN (4 × 10^9^ geq/mL) was 5.75 log (TCID_50_/mL).

To determine the infectious titer of CTV, a TCID_50_ assay based on viral RNA quantification was developed. Two 96-well plates were analyzed using the Spearman–Kärber algorithm, both giving the same infectious titer of 5.75 log (TCID_50_/mL). Figure 2 depicts one of the two plates.

### 3.2. Protein Expression of ORF2-Encoded Protein

**Figure 3:**
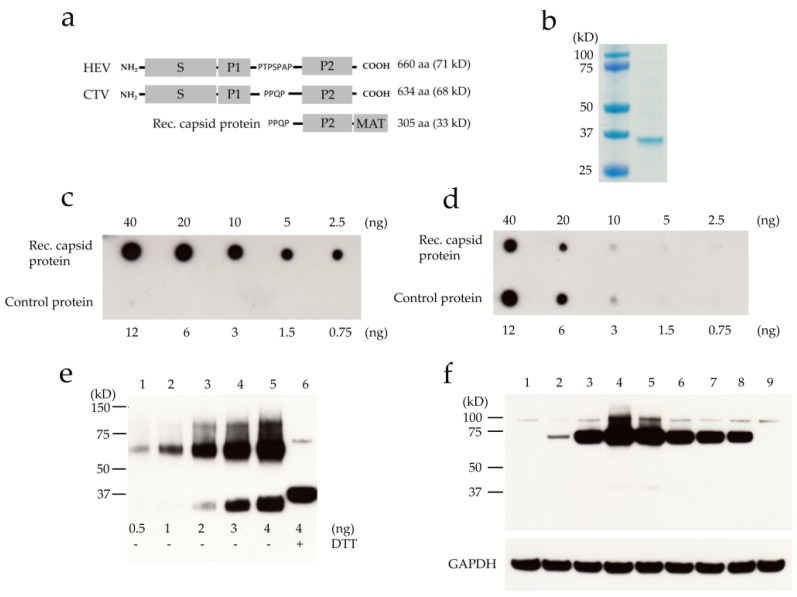
Generation of an antibody directed against the ORF2-encoded capsid protein. (**a**) Schematic representation of hepatitis E virus (HEV) and CTV ORF2-encoded protein domains and the region selected for antibody generation; (**b**) Reducing SDS-PAGE analysis of the affinity purified rec. capsid protein by staining with colloidal Coomassie stain; Dot blot detection of rec. capsid protein and metal affinity tag (MAT) tagged control protein using (**c**) anti-capsid protein antibody and (**d**) anti-MAT antibody; (**e**) Western blot analysis of the recombinant capsid protein at different concentrations (lanes 1–5) and treated with 100 mM Dithiothreitol (DTT) (lane 6); (**f**) Western blot detection of ORF2-encoded protein expression in infected RTGill-W1 cells over a time period of 12 days, using anti-capsid protein antibody; lanes 1 through 6 represent the respective days post-infection (pi); lane 7 represents day 8 pi; lane 8 represents day 12 pi; lane 9 was used as a negative control, representing non-infected RTGill-W1 cells twelve days in culture. Anti-GAPDH was used as a loading control.

In addition to the quantitation of viral RNA, the kinetics of ORF2 protein expression during the infection were examined. For this purpose, an antibody against the ORF2-encoded capsid protein was generated. The crystal structure of the HEV virus-like particle revealed that the P2 domain—located at the C-terminus of the protein—is on the surface of the capsid, and therefore accessible to antibodies [24]. Structural similarities were revealed upon comparing the structures of the CTV and HEV ORF2-encoded protein, such as the proline-rich hinge region, which appears to link the P2 domain with the P1 domain, as reported in HEV (depicted in Figure 3a). Considering the structural similarities with HEV and in silico predictions, which located the P2 region of CTV on the capsid surface, the c-terminal domain of the ORF2-encoded protein was selected for expression.

SDS-PAGE analysis indicated that the expressed protein was present in the insoluble fraction, allowing for inclusion body isolation. His-tag affinity purification successfully yielded a pure protein with the expected size (Figure 3b). The antibody was raised in a rabbit, purified via affinity chromatography, and tested for its ability to specifically detect the purified recombinant capsid protein. In a dot blot assay, the anti-capsid protein antibody could specifically detect the recombinant capsid protein throughout all dilutions. In contrast, no significant background was observed with the control protein (VP1u-MAT of parvovirus B19, Figure 3c). A control anti-MAT antibody showed that comparable amounts of both proteins were spotted (Figure 3d). Western blot analysis showed that as little as 0.5 ng of recombinant capsid protein can be easily detected, and that the protein exists in monomer and dimer forms. Under reducing conditions, the monomer was the predominant form, banding slightly higher than the monomer in the non-reduced samples (Figure 3e). This deviation in migration is most likely due to the non-reduced monomer being in a more compact structure due to disulfide bridges forming between the cysteine residues.

Having demonstrated its specificity, the anti-capsid protein antibody was used to follow the kinetics of ORF2-encoded protein expression during the infection, shown in Figure 3f. RTGill-W1 cells were inoculated with CTV, and samples were collected daily. Freeze–thaw cycles were used to obtain the intracellular ORF2-encoded protein, which was then analyzed by Western blot. A distinct band corresponding to the predicted molecular weight of the capsid protein (68 kD) could be faintly detected starting from 2 days post-infection (pi), with a rapid increase in signal as the days progressed. From day three through twelve, a strong signal was observed, with maximum intensity on day four. The mock-infected sample remained negative. A faint unspecific band could be detected above the capsid protein band at around 90 kDa, with similar intensity in infected and non-infected samples.

### 3.3. Immunofluorescence Detection of ORF2-Encoded Protein and dsRNA

**Figure 4:**
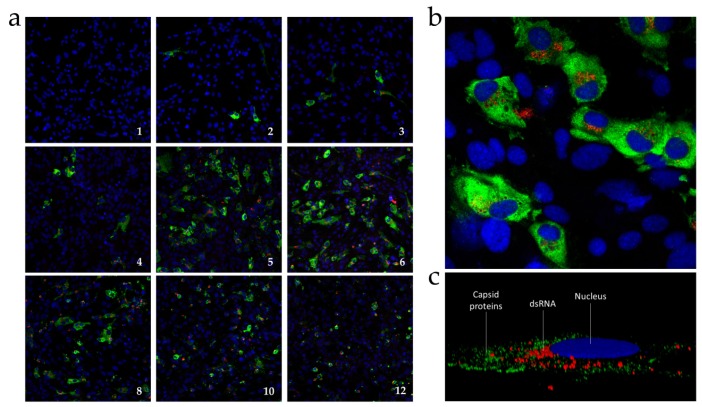
Confocal immunofluorescent analysis of infected RTGill-W1 cells. Cells were stained with anti-capsid protein antibody (green), anti-dsRNA antibody (red), and DAPI (blue); (**a**) Overview of the course of infection from day 1 to day 12 (20× magnification); (**b**) A 63× magnification of the infected cells on day 6; (**c**) Three-dimensional reconstruction using ZEN software from Zeiss microscopy.

To determine the percentage of infected cells and the subcellular localization of the ORF2-encoded protein, the viral infection was examined by immunofluorescence and confocal microscopy. The expression of capsid protein was followed over time in RTGill-W1 cells by using the polyclonal anti-capsid protein antibody. In addition, we stained the dsRNA intermediate with a monoclonal anti-dsRNA antibody. The results showed that both antibodies were highly specific. Figure 4a shows a steady increase of ORF2-encoded protein expression and dsRNA accumulation in cells infected with CTV up to six dpi. The maximum signal was obtained on days 5 and 6, with approximately 20% of cells infected. The capsid protein signal appears to be spread fairly uniformly across the cytoplasm, whereas the dsRNA signal appears to be more localized, often in proximity to the nucleus (Figure 4b) and organized in vesicle-like structures (Figure 4c). Between days 8 and 12, the capsid protein signal becomes less intense, and the cytoplasm appears reduced in size and more rounded, resembling the initiation of apoptosis (Figure 4a).

### 3.4. Association of CTV with Lipids

**Figure 5:**
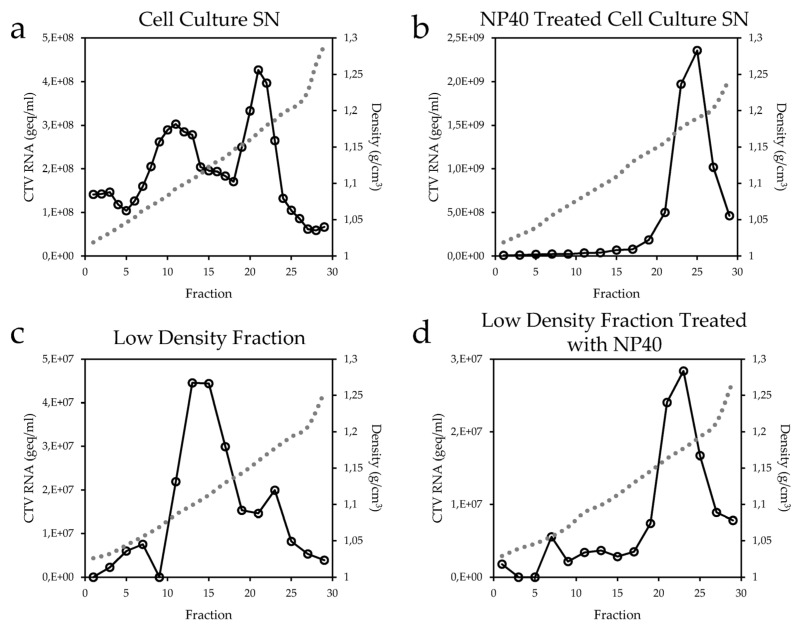
CTV produced in cell culture is associated to lipids. CTV RNA (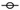) was quantified by RT-qPCR and the density (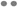) of each fraction was measured by refractometry. (**a**) Iodixanol density gradient of CTV from cell culture SN; (**b**) Treatment of cell culture SN with 1% NP40; (**c**) Fractions 10 and 11 of the low-density fraction from (a) were pooled and re-centrifuged; (**d**) The same fraction used for (c) was pretreated with 1% NP40 and re-centrifuged.

HEV has been shown to be associated to lipids in cell culture SN and in serum. In order to verify whether CTV produced in cell culture is also lipid-associated, cell culture SN of infected RTGill-W1 cells was collected, and the progeny particles resolved by equilibrium sedimentation in isotonic iodixanol gradients. Fractions were collected, and the presence of CTV was quantified by RT-PCR. Two populations of CTV were observed upon analysis of iodixanol gradients. One population banded between 1.07 and 1.11 g/cm^3^, and the other banded between 1.15 and 1.20 g/cm^3^ (Figure 5a). This difference in density—similar to that reported for hepatitis A virus and HEV—suggests that the less dense population is lipid-associated. Only the denser peak banding between 1.15 and 1.19 g/cm^3^ was detectable when the cell culture SN was pretreated with 1% NP40 (Figure 5b). This peak corresponds to the buoyant density of the high-density fraction in cell culture SN (Figure 5a). In order to confirm the delipidation by NP40 treatment, the peak of the low-density fraction in Figure 5a was centrifuged again without any pretreatment (Figure 5c) and with NP40 pretreatment (Figure 5d).

## 4. Discussion

Hepatitis E virus (HEV) is a causative agent of hepatitis worldwide and an emerging concern in industrialized countries. HEV research has been particularly hindered because the virus is difficult to grow in cell culture. Although some systems have been described [23], they are restricted to few HEV isolates and cell lines, the virus titers are typically low, and they require extensive incubation times. For these reasons, only a few aspects of the infectious cycle of the virus have been revealed so far. Therefore, an HEV-related virus from the *Hepeviridae* family that can consistently replicate in cultured cells would be an excellent tool to facilitate and complement studies with HEV. Of all members of the *Hepeviridae* family, cutthroat trout virus (CTV) is the only virus known to be non-pathogenic for humans and animals. The aim of these studies was to establish CTV as a robust surrogate virus to facilitate basic research with HEV. For this purpose, we have developed a novel cell culture system to propagate CTV to high titers within a short time and establish analytical tools to characterize the infection.

Previous cell culture systems of CTV in CHSE-214 cells reported viral titers between 10^7^ and 10^8^ geq/mL after 20 days in culture [6], which represented a modest improvement over other cell culture systems reported for HEV. We observed similar replication efficiencies of CTV in ZF4 cells, and less satisfactory replication in SOB-15 cells. Two rainbow trout epithelial cell lines—RTGill-W1 from the gill and RTL-W1 from the liver—were the best cell lines for CTV replication. These observations suggest that the gills—an organ in contact with surrounding water—might be the primary site of infection, and that (as for HEV) the liver is the secondary site of infection. Viral propagation in RTGill-W1 cells largely exceeded the titer obtained with CHSE-214 cells by up to 100 fold, and the incubation period was substantially reduced to 5–6 days. A similar result was reached with RTL-W1 cells with a slightly lower titer. This simple and highly efficient cell culture system contrasts with the difficulties associated with HEV cell culture systems, most of which have been reported as inefficient due to long periods of incubation and low titers. To our knowledge, the most efficient HEV cell culture system reported to date reached up to 3.9 × 10^8^ geq/mL in A549 cells at 10–20 days post-infection. However, six passages had to be performed in order to reach this titer [15].

In addition to a cell culture system, we developed analytical tools to follow CTV infection. A specific antibody directed against the capsid protein was used to follow the expression and intracellular distribution of the capsid protein during infection. The ORF2-encoded protein, with the expected size of 68 kDa, increases with a maximum signal on day 4, coinciding with the high level of CTV RNA. The slight decrease in signal on day 6 may be linked to viral egress, which peaked on day 6. During viral replication, the capsid proteins appeared spread throughout the entire cytoplasm, in contrast to the intermediate dsRNA species, which were predominantly located close to the nucleus and organized in large structures. This particular intracellular distribution in large clusters suggests that the viral replication occurs in membranous factories, as proposed for other plus-strand RNA viruses, such as the hepatitis C and Dengue virus [25].

The presence of dsRNA in the cells is a common signature linked to the replication of certain viruses. The dsRNA may originate from their genomes (viruses with dsRNA genomes) or as an intermediate RNA species through the transcriptional processes. The foreign dsRNA is detected by cellular dsRNA sensors, which stimulate strong protective responses, such as the induction of dicer-related pathways, interferon, dsRNA-activated protein kinase, and oligoadenylate synthetase [26]. The accumulation of dsRNA intermediates observed during CTV replication and the abrupt halt in virus replication observed after 5–7 days without cytopathic effects suggest that dsRNA-induced antiviral mechanisms have been triggered [27]. Replication of HEV in cells results in a similar halt in replication, which was linked to the induction of interferon signaling. Replication was improved by treatment of the target cells with the cell permeable inhibitor BX795, which blocked the phosphorylation and activation of interferon regulatory factor 3 (IRF3), leading to suppression of interferon-stimulated genes (ISGs) [28]. It has been demonstrated that CTV can also induce interferon-like activity in cultures of AK leucocytes [5]. Therefore, it is expected that interfering with innate immunity may lead to a further improvement of CTV replication.

Even though viruses are known to either be enveloped or non-enveloped, there seem to be viruses that blur the lines between these two groups. Hepatitis A virus (HAV), for example, has been shown to be enveloped when produced in cell culture [29,30]. Similarly, it was reported that HEV released from infected cells has a lipid envelope [31,32]. The presence of an envelope in HEV depends on the environment the virus encounters. HEV particles in feces do not possess an envelope, in contrast to those in cell culture SN and in blood, which are lipid-associated [33]. Ultracentrifugation through iodixanol density gradients revealed that CTV particles released from infected cells appear as lipid-associated as well as naked particles. Whether the lipid association occurs by utilizing the cytoplasmic endosomal sorting complexes required for transport (ESCRT) machinery (as with HEV [4]) or at the cell surface still requires investigation. The origin of the non-enveloped particle progeny in cell culture SN is unclear. Several possibilities exist, including partial cell lysis causing non-enveloped viruses to be released, interactions of the released enveloped virus with factors on host cellular membranes or with centrifugation tubes leading to a loss of the lipid envelope, or an alternative lipid-independent egress mechanism. Nevertheless, the fact that CTV—like HEV—is released from cells associated to lipids suggests that the acquisition of an envelope is a common strategy of the hepevirus family, and further validates CTV as an ideal model to investigate different aspects of the biology of these viruses.

In conclusion, our study describes the establishment of a cell culture system to replicate CTV, and the development of analytical tools to study the infection. The efficient cell culture method—which is the most robust achieved with a hepevirus—will be a promising tool to provide insight into the mechanisms of infection of *Hepeviridae* in general, and HEV in particular.
